# Chéilite tuberculeuse révélant une tuberculose pulmonaire

**DOI:** 10.11604/pamj.2016.24.176.9862

**Published:** 2016-06-30

**Authors:** Myriem Bricha, Hajar Slimani, Sanae Hammi, Jamal Eddine Bourkadi

**Affiliations:** 1 Service de Pneumo-phtisiologie, Hôpital Moulay Youssef, CHU Rabat, Maroc; 2 Service de Pneumo-phtisiologie, CHU Tanger, Maroc

**Keywords:** Chéilite, lèvre, cavité buccale, tuberculose, Cheilitis, lip, oral cavity, tuberculosis

## Abstract

La tuberculose de la cavité buccale reste rare. Elle admet un polymorphisme clinique et pose avant tout un problème de diagnostic. Nous rapportons le cas d'un homme de 42 ans présentant une chéilite tuberculeuse. L’intérêt de cette présentation est d’attirer l’attention sur la tuberculose que l’on peut retrouver de façon exceptionnelle dans certaines localisations, comme la lèvre buccale.

## Abstract (en)

Tuberculosis of the oral cavity is rare. It is associated with clinical polymorphism and poses above all a diagnostic problem. We report the case of a 42-year-old male patient with tubercular cheilitis. This study aims to focus attention on tuberculosis that can be detected exceptionally in specific locations, such as the oral lip.

## Introduction

La tuberculose est un problème majeur de santé publique, c’est une maladie pouvant se présenter selon un large spectre de manifestations pulmonaires et / ou extra-pulmonaire [1]. Cependant la tuberculose de la cavité buccale reste rare. Elle admet un polymorphisme clinique et pose avant tout un problème de diagnostic. L’étude bactériologique et histopathologique tient un rôle important, permettant de préciser la nature tuberculeuse des lésions. L’évolution est favorable sous traitement médical bien codifié.

## Patient et observation

**Figure 1:**
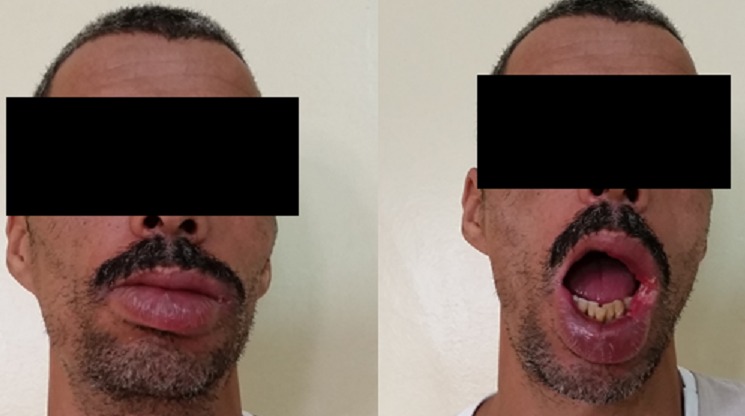
Photographie montrant une tuméfaction et ulcération de la lèvre chez un patient atteint de tuberculose labiale

Nous rapportons l´observation médicale d´un patient âgé de 42 ans, marocain, agriculteur de profession, sans habitudes toxiques, sans antécédents particuliers présentant depuis 4 ans un gonflement progressif de la lèvre inférieure qui s’étend vers la lèvre supérieure associé à une ulcération de la commissure labiale gauche (Figure 1). La symptomatologie n’a pas été accompagné de fièvre ni de manifestations allergiques. Il n´y avait aucune anomalie à l´examen systémique notamment l’hygiène bucco-dentaire était satisfaisante. L’examen local révèle une lèvre inférieure augmentée de volume avec des fissures. La surface de la muqueuse est oedémaciée. Le gonflement est étendu à la lèvre supérieure. La commissure labiale gauche est ulcérée. La muqueuse nasale, le plancher de la gorge et la langue étaient normaux. Ces lésions sont rebelles à tout traitement antibiotique ou antihistaminique. L’étude anatomopathologique de la biopsie de la muqueuse labiale montre une réaction inflammatoire chronique constituée de granulome épithélio-giganto-cellulaire. La radiographie thoracique montrait des opacités hétérogènes au niveau des deux tiers supérieurs. Il n´y avait pas d’adénopathie hilaire. La recherche de BAAR dans les crachats est revenue positive.

**Figure 2:**
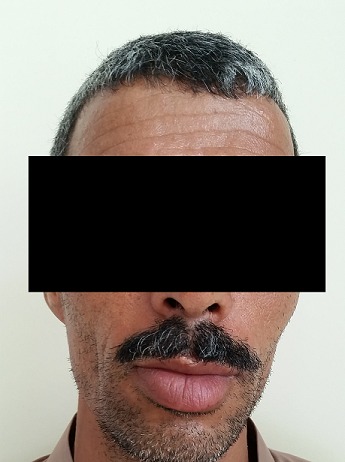
Photographie montrant une amélioration clinique après deux mois de traitement antibacillaire

Nous avons retenu le diagnostic de tuberculose pulmonaire avec chéilite granulomateuse et le traitement anti-tuberculeux de quatre médicaments (isoniazide, rifampicine, éthambutol et pyrazinamide) a été démarré et l’évolution était marquée par la régression de la tuméfaction et de l’ulcération (Figure 2).

## Discussion

La Chéilite granulomateuse est une inflammation granulomateuse avec infiltration de cellules mononucléaires des lèvres et de la peau péri-orale qui se présente comme un gonflement indolore locale diffuse des zones touchées. Elle peut être secondaire à de nombreuses conditions, y compris la maladie de Crohn, la sarcoïdose et les infections telles que la tuberculose [2]. Bien que les manifestations buccales de tuberculose ont été rapportées, elles sont rares, elle représente seulement 2-6% des cas de tuberculose extrapulmonaire et 0,1-1% de toutes les présentations cliniques de la TB [1]. L’atteinte buccale est une de ces localisations rares. Sa fréquence varie de 0,05 à 0,5% [3]. Les lésions buccales de la tuberculose peuvent être primaires ou secondaires. Dans la maladie primaire, la bouche est le site initial de l´infection, il est rare et touche surtout les jeunes plutôt que les adultes plus âgés [1]. La muqueuse buccale intacte possède une résistance naturelle à l’infection [4] en raison de la présence d´enzymes salivaires, des anticorps circulants, et de l´épithélium et de l´architecture du tissu conjonctif. Toute rupture ou la perte de cette barrière naturelle, qui peut être le résultat d´un traumatisme, les conditions inflammatoires, extraction d´une dent ou une mauvaise hygiène buccale, peuvent fournir une voie d´entrée pour la mycobactérie. Cependant, l´inoculation directe de la tuberculose orale primaire peut se produire en cas d´ulcères, de fissures ou de gonflement de la muqueuse [4]. La TB orofaciale secondaire est généralement une complication d’une tuberculose d´un autre site et peut donc être due à l´inoculation par voie orale avec des expectorations infectées ou due à la dissémination hématogène éventuellement des mycobactéries [1], elle est plus fréquente [4]. Les lésions buccales de la tuberculose ne sont pas spécifiques dans leur présentation et sont souvent négligés par les cliniciens ou mal diagnostiquée comme des aphtes ou d’origine néoplasique. Par conséquent, il est important de penser à la tuberculose en cas d’ulcères buccaux [5].

## Conclusion

L’originalité de notre travail est la localisation de la tuberculose au niveau de la lèvre buccale qui constitue une localisation rare et sa survenue chez un sujet adulte en absence de facteur favorisant. A travers ce travail, on souligne l’importance d’un diagnostic précoce d’une lésion tuberculeuse et la recherche d’un site primaire de la maladie même en absence de signes d’appel.

## References

[1] Ilyas S, Chen F, Hodgson T, Speight P, Lacey C, Porter S (2002). tuberculosis: a unique cause of lipswellingcomplicating HIV infection. HIV Med.

[2] Bhattacharya M, Rajeshwari K, Sardana K, Gupta P (2009). Granulomatouscheilitissecondary to tuberculosis in achild. J Postgrad Med.

[3] Koffi S, Kouassi A, Faye-Kette H, Kouassi-M'bengue A, Ahui J, Aka-Danguy E (2008). Tuberculose de la muqueuse buccale chez un patient immunodéprimé par le VIH-1. Med Mal Infect.

[4] Gupta A, Narwal A, Singh H (2014). Primary Labial Tuberculosis: A Rare Presentation. Ann Med Health Sci Res.

[5] Tovaru S, Costache M, Sardella A (2008). Primary oral tuberculosis: a case seriesfromBucharest, Romania. Oral Surg Oral Med Oral Pathol Oral Radiol Endod.

